# An Interpretable Early Dynamic Sequential Predictor for Sepsis-Induced Coagulopathy Progression in the Real-World Using Machine Learning

**DOI:** 10.3389/fmed.2021.775047

**Published:** 2021-12-03

**Authors:** Ruixia Cui, Wenbo Hua, Kai Qu, Heran Yang, Yingmu Tong, Qinglin Li, Hai Wang, Yanfen Ma, Sinan Liu, Ting Lin, Jingyao Zhang, Jian Sun, Chang Liu

**Affiliations:** ^1^Department of Hepatobiliary Surgery, The First Affiliated Hospital of Xi'an Jiaotong University, Xi'an, China; ^2^Department of SICU, The First Affiliated Hospital of Xi'an Jiaotong University, Xi'an, China; ^3^School of Mathematics and Statistics, Xi'an Jiaotong University, Xi'an, China; ^4^Department of Clinical Laboratory, The First Affiliated Hospital of Xi'an Jiaotong University, Xi'an, China; ^5^Biobank, The First Affiliated Hospital of Xi'an Jiaotong University, Xi'an, China

**Keywords:** SIC, sepsis-associated DIC, irregular time-series data, early real-time prediction, machine learning

## Abstract

Sepsis-associated coagulation dysfunction greatly increases the mortality of sepsis. Irregular clinical time-series data remains a major challenge for AI medical applications. To early detect and manage sepsis-induced coagulopathy (SIC) and sepsis-associated disseminated intravascular coagulation (DIC), we developed an interpretable real-time sequential warning model toward real-world irregular data. Eight machine learning models including novel algorithms were devised to detect SIC and sepsis-associated DIC 8*n* (1 ≤ *n* ≤ 6) hours prior to its onset. Models were developed on Xi'an Jiaotong University Medical College (XJTUMC) and verified on Beth Israel Deaconess Medical Center (BIDMC). A total of 12,154 SIC and 7,878 International Society on Thrombosis and Haemostasis (ISTH) overt-DIC labels were annotated according to the SIC and ISTH overt-DIC scoring systems in train set. The area under the receiver operating characteristic curve (AUROC) were used as model evaluation metrics. The eXtreme Gradient Boosting (XGBoost) model can predict SIC and sepsis-associated DIC events up to 48 h earlier with an AUROC of 0.929 and 0.910, respectively, and even reached 0.973 and 0.955 at 8 h earlier, achieving the highest performance to date. The novel ODE-RNN model achieved continuous prediction at arbitrary time points, and with an AUROC of 0.962 and 0.936 for SIC and DIC predicted 8 h earlier, respectively. In conclusion, our model can predict the sepsis-associated SIC and DIC onset up to 48 h in advance, which helps maximize the time window for early management by physicians.

## Introduction

Sepsis is a lethal disease caused by a dysregulated host response in an infected state (1). Septic-induced organ dysfunction is a major cause of sepsis high mortality (2). Among these, coagulation dysfunction is a pervasive complication of sepsis, occurring in 50–70% of sepsis patients, while approximately 35% of patients proceed to disseminated intravascular coagulation (DIC) (3). Sepsis-induced coagulopathy (SIC) mortality reaches to 23.1% (4), while the mortality rate of sepsis-associated DIC is more than twice that of simple sepsis patients (5, 6). According to the SIC scoring system proposed by the DIC subcommittee of International Society on Thrombosis and Haemostasis (ISTH) in 2017, sepsis patients easily meet the SIC diagnostic criteria (7). Sepsis-associated DIC was diagnosed by the two-step sequential approach of SIC and ISTH overt-DIC criteria, which is a late-phase coagulation disorder that should be detected early (8). Currently, DIC diagnosis does not have a gold standard. Physicians diagnose DIC according to the primary disease, clinical manifestations, and laboratory tests. However, the clinical signs and symptoms of DIC appear slowly and the manifestations are complex and varied, resulting in a time lag in the clinical diagnosis of DIC, which places the patient in a treatment-refractory phase when sepsis-associated DIC is clinically determined (9). In addition, studies have shown that anticoagulation is ineffective in both sepsis and SIC patients, but effective in sepsis-induced DIC patients (10–12). Hence, early recognition of sepsis-associated DIC is more important than SIC, while there are currently no studies on sequential prediction of sepsis-associated DIC after SIC alerts. Therefore, it is imperative to establish a model for early sequential real-time prediction of SIC and sepsis-associated DIC.

The prevalence of electronic health records (EHRs) and the upsurge of artificial intelligence (AI) provide opportunities for clinical medical research (13). Studies have shown that machine learning-based models outperform traditional clinical scoring and human expert systems in the diagnosis, treatment, and prognosis prediction of clinical diseases (14, 15). However, current clinical prediction studies are mainly static and lack real-time prediction studies. Real-time prediction models dynamically predict the onset of disease within a sliding time-window by continuously updating clinical data. From the clinical dynamic treatment perspective, real-time predictive models would better fit the clinical applications (16). In addition, the variability of primary diseases, comorbidities and severity of conditions in ICU patients leads to sparse and irregular clinical data in terms of sampling time and dimensions (17). To accommodate irregular time series data, the existing standard models such as eXtreme Gradient Boosting (XGBoost) (18) and Recurrent Neural Network (RNN) (19) decompose time into continuous, non-overlapping uniform intervals, known as temporal discretization (20). This enables the standard models to act on fixed dimensional vectors (regular data). However, this approach lacks the continuity principle and can lead to undesirable results when applied to irregular medical time-series data (21). Altogether, it is necessary to develop a model that is specifically designed to handle sparse irregular time series data in the real clinical world to achieve real-time accurate predictions at arbitrary time points.

**Figure 1:**
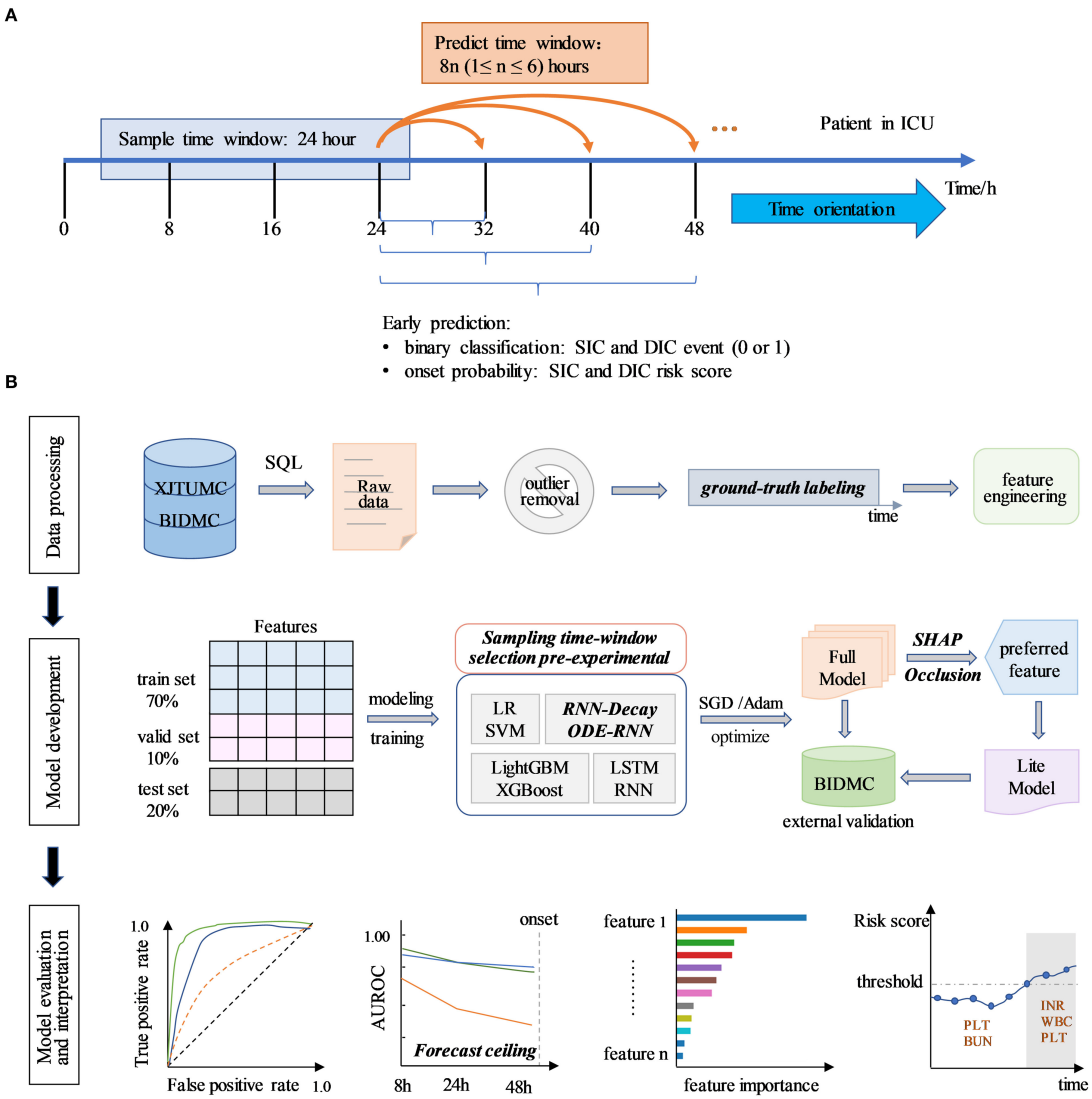
Study design and model development overview. **(A)** Research application design profile. Study developed an early real-time prediction model to dynamically predict disease progression of sepsis patients after admission to ICU, and to output whether the patient will suffer from SIC and sepsis-associated DIC and the risk score for their onset in future periods. The model feeds into the information obtained from a continuous 24 h sampling time-window and outputs the disease status for the next 8*n* h in real-time. The x-axis indicates the time since admission. **(B)** Model development overview. The research process comprises to data processing, model development, model evaluation, and interpretation. The data processing process includes raw data extraction, outlier processing, coagulation status annotation, and feature engineering. The model development stage included feature set split, sampling time-window selection pre-experimental, model construction, and training. We built eight models, trained and selected the Full model with good performance on the internal validation set by SGD or Adam optimization. The Lite models were developed by selecting easily accessible and important features through SHAP and Occlusion analysis. Models were validated at BIDMC for external validation. The model evaluation and interpretation section include the assessment of model predictive performance, interpretation of model outputs, individualized disease trajectory prediction, etc.

In summary, we aim to help physicians to identify patients at high risk of SIC and sepsis-associated DIC early, especially those who progress to DIC after SIC, as well as improve existing machine learning models to enable arbitrary time-point prediction on real-world irregular data. To achieve this, an interpretable early real-time sequential warning predictor will be developed that contributes to early personalized treatment and reasonable administration. The overview of the study design and model development was shown in Figure 1.

## Methods

### Study Cohort and Design

This is a multicenter retrospective cohort study. Research data were obtained from two medical centers, the Xi'an Jiaotong University Medical College (XJTUMC) and Beth Israel Deaconess Medical Center (BIDMC). Structured query language (SQL) was used to obtain eligible patient data for the period from January 1, 2013 to December 1, 2018 in XJTUMC and from 2001 to 2012 in BIDMC, respectively. The XJTUMC data were obtained from the Center's Biobank and the BIDMC data were obtained from the Medical Information Mark for Intensive Care (MIMIC-III) database (22). The study was reviewed by the Ethics Committee of the First Affiliated Hospital of Xi'an Jiaotong University, and all data were deidentified.

**Figure 2:**
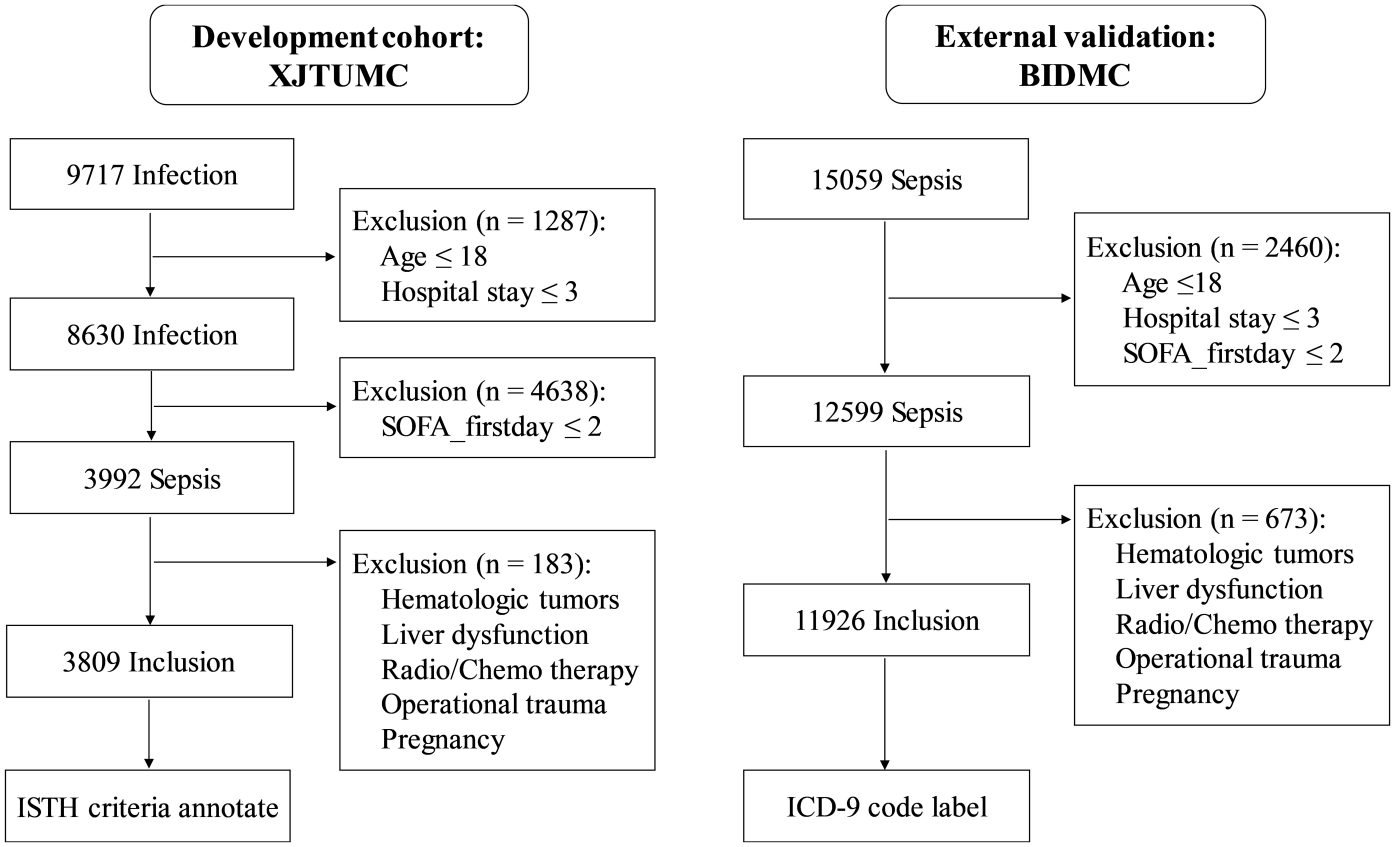
The diagram of study patient enrollment. In XJTUMC, coagulation status annotation was based on SIC and ISTH-overt DIC criteria. In BIDMC, DIC were labeled based on ICD-9. ICD, International Classification of Diseases.

The enrollment process was shown in Figure 2. The inclusion criteria were as follows: (1) sepsis was diagnosed within 24 h of admission based on Sepsis 3.0 criteria; (2) the age was not <18 years; and (3) the duration of hospitalization was not <3 days. The exclusion criteria were as follows: (1) patients with DIC onset within 24 h of admission; (2) patients were affected by hematologic tumors (leukemia, lymphoma, etc.); (3) patients suffering from cirrhosis, acute liver failure, with liver function up to Child C; (4) patients treated with radiotherapy or chemotherapy; (5) patients with admission diagnosis of combat trauma, traumatic coagulopathy; (6) patients with pregnancy or perinatal complications.

### Data Preprocessing

We initially obtained a total of 174 laboratory features in XJTUMC and 259 laboratory features in BIDMC. Subsequently, we performed the process of merging identical variables (e.g., HGB with different units of g/L or g/dl, HGB in blood count and blood gas test, etc.), eliminating irrelevant variables (e.g., hepatitis antibody quantification, blood drug concentration, etc.), and counting the frequency of variable detection. We removed indictors that were completely missing and detected <1% of indicators at all-time points. In addition, we performed the unit conversions for the XJTUMC laboratory test variables, in order to maintain consistency with BIDMC. Ultimately, under the guidance of the laboratory physicians, we identified 99 features at XJTUMC and 72 features at BIDMC. Of these, the 72 features at BIDMC were common features for both medical centers. The missing information for both medical centers were shown in the Supplementary Figure 1 (Supplementary File 1). And the clinical reportable ranges for each identified variable was shown in Supplementary File 2. All variables were initialized by the min-max normalization algorithm.

Clinical sequence data are sparse and irregular, manifested by the presence of a large amount of missing data. Whereas, the pattern of missing data contains important information, such as the correlation between the frequency of a certain marker test and the severity of the disease. Therefore, in this study, we do not deal with missing data. Instead, we use the modeling of missing information to identify the role of missing patterns in prediction, which enhances the model prediction effectiveness. In brief, it is modeled by learning to characterize the missing and hidden information from the time-series data, which is then further introduced into the model network. The multivariate time series *X* = {*x*_1_, *x*_2_, …, *x*_*T*_} is the observations at time *T*. $x_{t}\in R^{D}$represents the observations at time *t* for all variables. *x*_*t*_ contains *D* features $\left\{x_{t}^{1},x_{t}^{2},x_{t}^{D}\right\}$ and $x_{t}^{d}$ represents the *d*-th feature variable of *x*_*t*_. *s*_*t*_ denotes the timestamp of observation *x*_*t*_. We assume that the timestamp of the first observation is 0 (*s*_*t*_ = 0), the time interval between different timestamps may be the same or different. Δ indicates adjacent time steps for each variable. To provide an efficient representation of the missing values, we introduce the mask vector $m_{t}^{d}=\left\{0,\;1\right\}$ to represent the missing variables in *x*_*t*_ at time *t*. Some features are missing continuously over a period of time, and $\delta_{t}^{d}$ is defined to represent the time interval between the last observation and the current timestamp. To be more specific, we have:


(1) $$m_{t}^{d}\;=\{\begin{array}{cc} 1, & if\;x_{t}^{d}\;is\;observed\\ 0, & otherwise \end{array}$$

(2) $$\delta_{t}^{d}\;=\{\begin{array}{cc} s_{t}-s_{t-1}+\delta_{t-1}^{d} & t>1,\;m_{t-1}^{d}=0\\ s_{t}-s_{t-1} & t>1,\;m_{t-1}^{d}=1\\ 0 & t=1 \end{array}$$

Thus, a dynamic feature of the input would be represented as $X_{t}=\left(x_{t}^{d},m_{t}^{d},\Delta ,\delta_{t}^{d}\right)$. In the later section, the missing information will be introduced into the model for subsequent processing when improving the algorithm model.

### Ground-Truth Labels Using ISTH

We defined all three disease states according to SIC and ISTH overt-DIC criteria. The details of the disease status annotation criteria can be found in Supplementary Table 1 (Supplementary File 1). New data were not available for all 8 h time-window of the day because the patient's laboratory tests were irregular. If there are no updated data available for labeling in an 8 h time-window, then the forward interpolation method is used for labeling based on the labels before and after that time-window. More specific details were shown in Supplementary Figure 2 (Supplementary File 1).

### Continuous Models for Irregular Time Series

**Figure 3:**
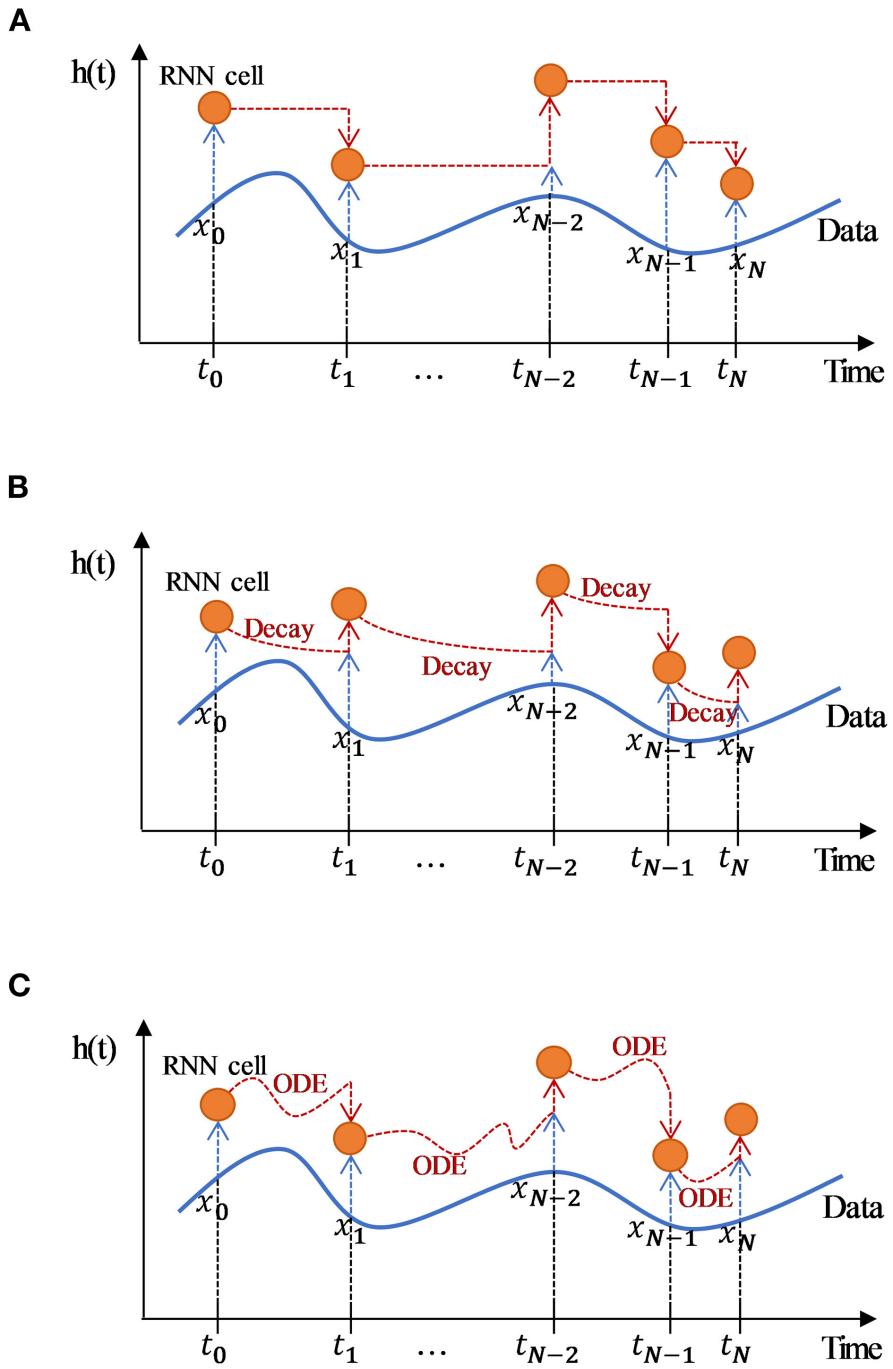
Architecture diagram of different RNN-based models for processing irregular time-series data**. (A)** Diagram of the standard RNN model processing irregular time-series data. The standard RNN transmits the hidden layer information forward at a fixed time interval without processing the hidden layer information; **(B)** Diagram of RNN-Decay model processing irregular time-series data. Based on the RNN framework, RNN-decay adds temporal decay derived from time intervals to the latent space in order to indirectly process irregular series; however, the longer the time interval, the weaker the information conveyed. **(C)** Diagram of ODE-RNN model processing irregular time-series data. The ODE-RNN directly modeled the unequally spaced raw data, and propagated a latent variable along the time interval using the Neural ODE to solve the latent layer information that corresponds to the irregular timestamp; this allows the arbitrary time-point data to be processed and guarantees temporal continuity. The time series *T* = {*t*_0_, *t*_1_, …, *t*_*N*_} is the observation timestamps, *X* = {*x*_0_, *x*_1_, …, *x*_*N*_} represents the observations at time *t* for all variables, *h*(*t*) is the latent space state corresponding to timestamp *t*. Orange circles indicate an RNN unit. Blue and black dashed lines with arrows indicate the shallow and latent features of the input RNN, and orange dashed lines with arrows indicate data from the previous RNN unit that has been processed in a different way and then fed into the next RNN unit.

The study compared two methods for dealing with irregular time series. The first is the temporal discretization approach, in which the standard RNN model is a typical model. In the second approach, we tackle the irregular time-series problem by modeling the missing information. Based on the standard RNN model, we introduced a decay mechanism for modeling missing information by referencing Che et al.'s study (21), to eventually develop the RNN-Decay model. In addition, we developed the Ordinary Differential Equations-Recurrent Neural Networks (ODE-RNN) model by directly modeling the unequally spaced raw data referring to the work of Yulia et al. (23). The model architecture diagrams were shown in Figure 3. The model inputs are derived from the extraction of missing patterns of time series information from “Data Processing” described in the above section.

### Model Development

To select the appropriate sampling time length, we chose 8, 24, and 48 h sampling time-window to perform pre-experiments. The results were presented in the Supplementary Table 2 (Supplementary File 1). Finally, we set the sampling time-window to 24 h and the sliding time-step to 8 h. Then, we developed several state-of-the-art models that are widely used as follows: (1) Classic machine learning models: Logistic regression (LR) (24) and support vector machines (SVM) (25), which are the most commonly used algorithms in existing research; (2) Enhanced machine learning models: gradient boosting machine (LightGBM) (26) and XGBoost (27), which are widely regarded as the best algorithm for data prediction and are adopted by many competition winning models in the field of machine learning; (3) Classic deep learning models: RNN (21) and long short-term memory network (LSTM) (28), which are the most commonly chosen deep learning models in time-series data, which have shown excellent performance in several time series studies; (4) Improved deep learning models: RNN-Decay and ODE-RNN. The detailed method was described in Supplementary File 1. After evaluating the performance, we finally chose the highest-performing XGBoost model as our predictor.

### Model Evaluation

We random divided the XJTUMC data into a train set (70%), a valid set (10%), and a test set (20%). The BIDMC data ware used as the external validation set to evaluate generalization ability of the model. Parameter optimization were performed by Stochastic Gradient Descent (SGD) or the Adam algorithm. The area under the receiver operating characteristic curve (AUROC), the area under the precision-recall curve (AUPRC) and the F1-score were used as model evaluation metrics. Test samples were resampled 1,000 times using bootstrapping to calculate 95% confidence intervals.

### Model Interpretation

In this study, we interpreted the machine learning model results using the Shapley Additive exPlanations (SHAP) algorithm (29) and the deep learning model results using Occlusion analysis (30).

### Statistical Analysis

Baseline data are skewed and expressed as the median and interquartile range (IQR). Non-parametric tests were used for statistical tests. *P*-values < 0.001 were considered statistically significant. Navicat Premium (12.1.22), Pytorch (1.7.0), and Python (3.7.6) with Numpy (1.18.5), Pandas (1.1.5), and Scikit-learn (0.23.2) formed the data-processing pipeline. All computational analyses were performed in the Computer Center of the School of Mathematics, Xi'an Jiaotong University.

## Results

### Study Baseline

A total of 9,717 infection patients in XJTUMC and 15,059 sepsis patients in BIDMC were initially included when applying ICD-9 codes and sepsis 3.0 criteria. After applying the exclusion criteria, 3,809 and 11, 926 patients were left. We then annotated the coagulation status of 3,809 XJTUMC sepsis patients by SIC and ISTH overt-DIC criteria, a total of 12,154 SIC status labels (positive: 8,909; negative: 3,246); and 7,878 overt-DIC status labels (positive: 3,051; negative: 4,827); were available in XJTUMC. Also, we selected 296 patients (1,210 status labels) who developed DIC during hospitalization at the BIDMC center using ICD-9 codes. The baseline characteristics of included patients at XJTUMC and BIDMC were shown in Table 1. For both XJTUMC and BIDMC, the median age of sepsis patients is above 60 years and the predominant cause of sepsis was respiratory system-derived infections. Also, 652/1,415 (46.1%) of SIC patients developed to DIC, while 652/679 (96%) of DIC patients fulfilled the SIC diagnosis.

**Table 1:** Baseline characteristics of included patients at XJTUMC and BIDMC.

	**XJTUMC (*n* = 3,809)**	**BIDMC (*n* = 11,926)**
Demographic		
Age (year), median [Q1, Q3]	63 [52,72]	69 [57,80]
Male, *n* (%)	2,388 (62.7)	6,481 (54.3)
Severity status at admission		
SOFA score, median [Q1, Q3]	3 [3, 4]	4 [5, 8]
Infection sources in sepsis, *n* (%)		
Respiratory system	1,702 (44.7)	4,957 (41.6)
Gastrointestinal system	1,435 (37.7)	3,194 (26.8)
Urinary system	5 (0.1)	382 (3.2)
Cardiac bloodstream system	24 (0.6)	728 (6.1)
Oncology cachexia related	567 (14.9)	1,077 (9.0)
Other	76 (2.0)	1,588 (13.3)
Outcome, median [Q1, Q3]		
Hospital stay (day)	7 [10, 15]	13 [7, 23]
Coagulation status		
SIC onset, *n* (%)	1,415 (37.1)^a^	Unknown
DIC onset, *n* (%)	679 (17.8)^a^	296 (2.5)^b^

*For infection sources in sepsis, respiratory system infections mainly cover the lung, trachea, bronchus, and chest related diseases; gastrointestinal system infections typically involve the esophageal, gastric, bowel, liver, spleen, and abdominal related disorders; urinary system infections include renal, ureteral, bladder, and urethra inflammatory diseases; cardiac bloodstream system comprise the cardiac, vascular, vascular catheter, and systemic infection related diseases; oncology cachexia is a variety of malignant diseases and cachectic manifestations; other infections consist of various inflammatory states of unknown etiology and brain diseases.*

a *Diagnosis based on SIC and ISTH-overt DIC criteria for coagulation status annotation.*

b *DIC diagnosis based on International Classification of Diseases-9th edition (ICD9) codes (ICD9 diagnosis code for DIC is 2866)*.

### Full Model Performance

**Figure 4:**
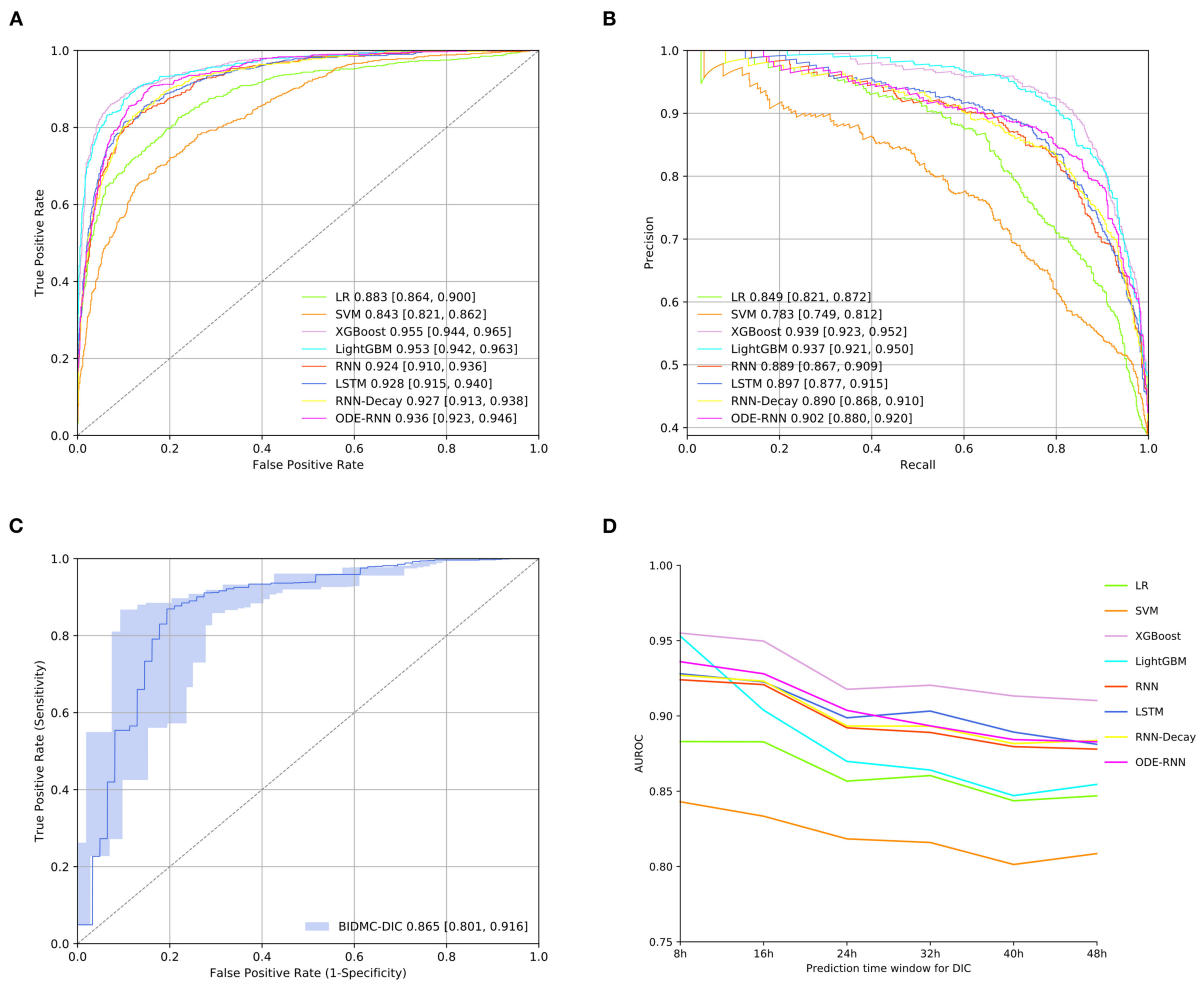
Full model performance evaluation. **(A)** Receiver operating characteristic curves of models for DIC prediction on the internal test set. **(B)** Precision-recall curve of candidate models for DIC prediction on the internal test set. **(C)** Receiver operating characteristic curves on the external test set. **(D)** The predictive performance of models at 8, 24, and 48 h ahead of sepsis-associated DIC onset. Precision was defined as the fraction of correctly alerted 8 h before the ground-truth event. Recall was defined as the proportion of any alarms 8 h prior to the ground-truth event. The lighter-colored intervals above and below the ROC and PR curves are 95% confidence intervals.

**Figure 5:**
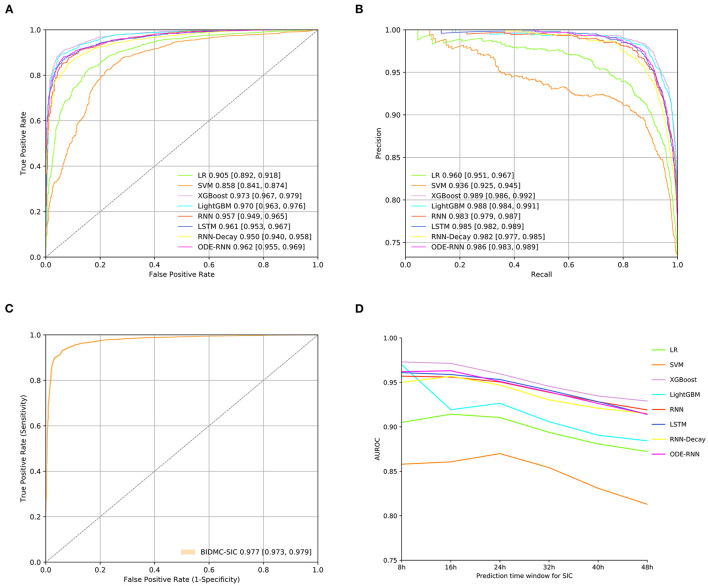
Full model performance evaluation for SIC prediction. **(A)** Receiver operating characteristic curves of models for SIC prediction on the internal test set. **(B)** Precision-recall curve of candidate models for SIC prediction on the internal test set. **(C)** Receiver operating characteristic curves on the external test set. **(D)** The predictive performance of models at 8, 24, and 48 h ahead of SIC onset.

The predictive performance of eight different models for early DIC onset were shown in Figure 4. Figures 4A,B showed that XGBoost produced the best prediction performance (AUROC: 0.955; AUPRC: 0.939) and was validated in LightGBM, followed by ODE-RNN (AUROC: 0.936; AUPRC: 0.902). However, ODE-RNN ensures continuity of model prediction, which is more suitable for clinical applications than XGBoost. Figure 4C revealed that the performance of the XGBoost on the BIDMC external validation set has decreased (AUROC: 0.865). Figure 4D provided prediction performance of the model at different prediction time-window, revealing that our model could detect the event as early as 48 h before the ground-truth. Figure 5 illustrated the prediction performance for SIC, showing similar results to DIC. Figures 5A,B showed that XGBoost provided the best prediction performance (AUROC: 0.973; AUPRC: 0.979). Figure 5C revealed that the performance of the XGBoost on the BIDMC external validation set has decreased (AUROC: 0.973). Figure 5D revealed that XGBoost could detect the event as early as 48 h before the ground-truth with AUROC reached 0.929. The detailed predictive performance of models at different prediction time-window was shown in Table 2. The model prediction performance decreases steadily as the prediction time-window extends. The XGBoost and ODE-RNN still maintain good prediction performance for SIC and DIC in the 48 h ahead of prediction time-window. Furthermore, we examined the early warning performance of the models on SIC and DIC with different alert thresholds at the 8 h prediction time-window, the results were shown in Supplementary Tables 3, 4 (Supplementary File 1). That allows clinicians to select different thresholds according to the characteristics of the different stages of disease development and treatment needs.

**Table 2:** Model performance at different prediction time-window for SIC and DIC prediction.

**Model-AUROC**	**SIC**			**DIC**		
**Prediction time-window**	**8 h**	**24 h**	**48 h**	**8 h**	**24 h**	**48 h**
LR	0.905	0.911	0.872	0.883	0.857	0.847
SVM	0.858	0.870	0.813	0.843	0.818	0.809
XGBoost	0.973	0.960	0.929	0.955	0.918	0.910
LightGBM	0.970	0.926	0.884	0.953	0.870	0.855
RNN	0.957	0.951	0.919	0.924	0.892	0.878
LSTM	0.961	0.953	0.914	0.928	0.899	0.881
RNN-decay	0.950	0.947	0.915	0.927	0.893	0.884
ODE-RNN	0.962	0.951	0.914	0.936	0.904	0.883

*SIC, sepsis-induced coagulopathy; DIC, disseminated intravascular coagulation; LR, logistic regression; SVM, support vector machines; LightGBM, light gradient boosting machine; XGBoost, eXtreme gradient boosting; RNN, recurrent neural network; LSTM, long short-term memory network; RNN-decay, recurrent neural networks-decay; ODE-RNN, ordinary differential equations-recurrent neural networks*.

### Model Interpretation

**Figure 6:**
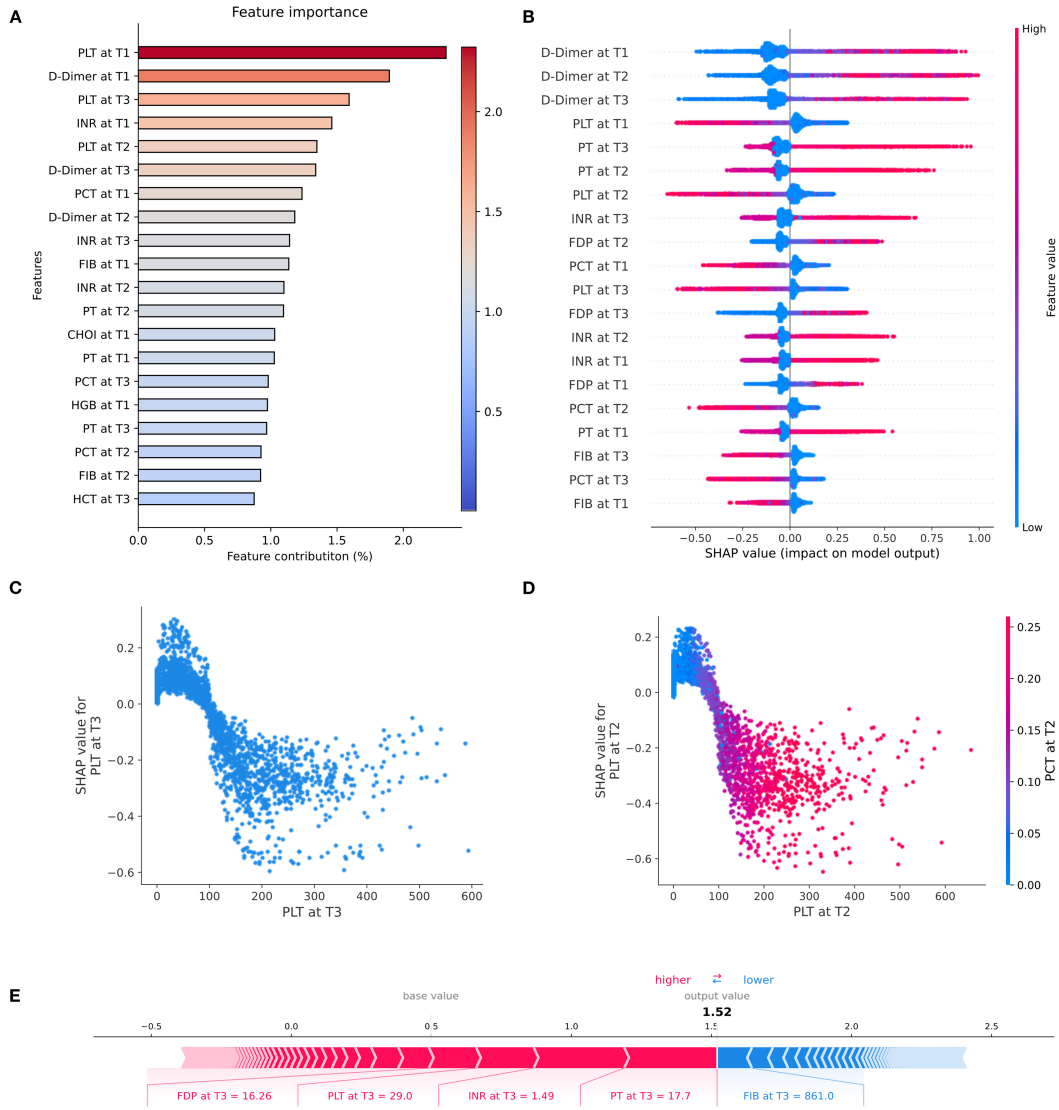
SHAP-based interpretation of model. **(A)** Overall feature importance of the top 20 features. **(B)** Beeswarm plot showing the impact of the top 20 features across all samples in the model. Beeswarm combines feature importance and feature effect, ranking the features by the sum of the SHAP over all samples (y-axis). In the plot, one row represents one feature and each dot represents the feature SHapley value for one sample, gray dots represent missing values, colors represent feature values (red high, blue low). Long tails indicate that features are extremely important for a particular patient. The x-axis represents the impact on the model output, with positive values pushing risk higher and negative values driving risk lower. **(C)** SHAP dependence plot showing predicted risk vs. feature value for PLT. The x-axis is the range of eigenvalues of PLT features and the y-axis is the shape-value of PLT features. It showed that when PLT was below 80, the shape-value was significantly increased with higher predicted risk. **(D)** SHAP interaction dependency plot describes the relationship between two interaction features and predicted risk. It showed the interaction between PLT and PCT, where when PCT is low, the corresponding PLT feature value is low, SHAP takes a high value and the model output risk increases. **(E)** SHAP force plot shows an interpretable example of single-sample feature prediction results. The base value is the predicted average of the all sample, and the output value is the predicted value of the current sample. Red indicates that the feature increases the risk, blue indicates that the feature decreases the risk. Longer feature bars indicate that the feature is contributing more.

To understand the contribution of the features to the model predictions, we interpreted the XGBoost predictions using Shapley values, which were presented in Figure 6. Figure 6A showed the top 20 features that contribute most to the model output; Figure 6B showed the impact of the top 20 features on all samples in the model. Shaply analysis identified that the most valuable features for DIC prediction were platelet (PLT), D-dimer, International normalized ratio (INR), plateletcrit (PCT), fibrinogen (FIB), fibrin degradation products (FDP). We further developed dependency plots to capture the non-linear correlation between a single significant feature and the predicted risk. As an example, Figure 6C showed that when PLT was below 80, the shape-value was significantly increased with higher predicted risk. Figure 6D showed the interaction between PLT and PCT, where when PCT is low, the corresponding PLT feature value is low, SHAP takes a high value and the model output risk increases. In addition, the SHAP force plot provides insight into the output risk and decision factors for specific samples. In the case of Figure 6E, the model predicts the sample at a high risk of a DIC event based on PT, INR, and PLT.

In addition, we interpreted the deep learning model ODE-RNN using occlusion analysis, as shown in Supplementary File 3. Occlusion analysis showed that a global absence of a single feature has small impact on the model output results for the ODE-RNN model. We also performed univariate and multivariate analyses of baseline data on the first day for different coagulation status groups, as detailed in Supplementary Tables 5, 6 (Supplementary File 1), suggesting the reliability of the machine learning approach.

**Figure 7:**
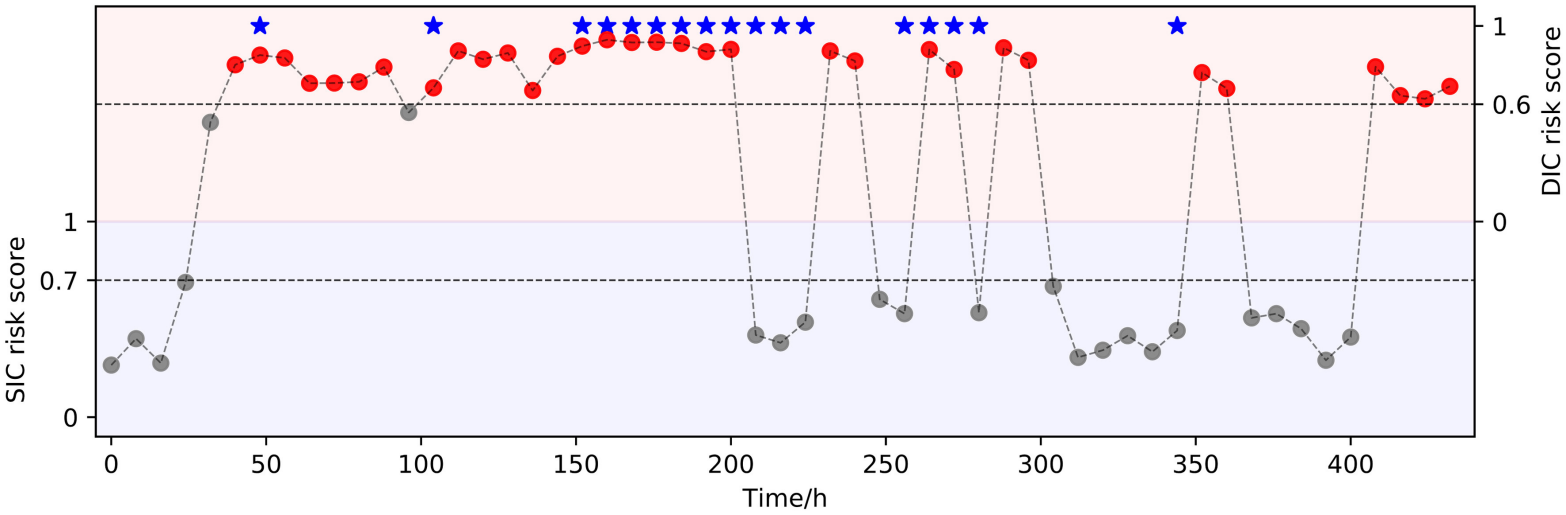
An interpretable real-time risk forecasting example. A 69-year-old male patient was hospitalized with the diagnosis of “Acute biliary pancreatitis, Obstructive jaundice.” The patient was diagnosed with SIC on the third day and sepsis-associated DIC on the fifth day of admission by the clinician. Our model predicted the disease progression earlier than the clinician. The predictions revealed that the most of sepsis-associated DIC events could be accurately predicted up to 8 h earlier. However, there were some sepsis-associated DIC events were still not predicted, mainly in the persistence of DIC onset, which might be related to clinical medicine interventions. The bottom of the graph is the SIC risk score and the upper is the DIC risk score. Red circles indicate sepsis-associated DIC events evaluated by the model. Red stars indicate true future predictions (meeting the ISTH overt-DIC criteria).

Figure 7 showed an example of real-time sequential prediction using our model on one patient. At each time point after the patient admission over 24 h, the model provides a real-time risk and uncertainty assessment of the future SIC and sepsis-associated DIC onset. This showed that the model could detect SIC and sepsis-associated DIC 48 h early, which is important for clinicians to take precautionary approaches ahead of the event.

### Lite Model Development

**Figure 8:**
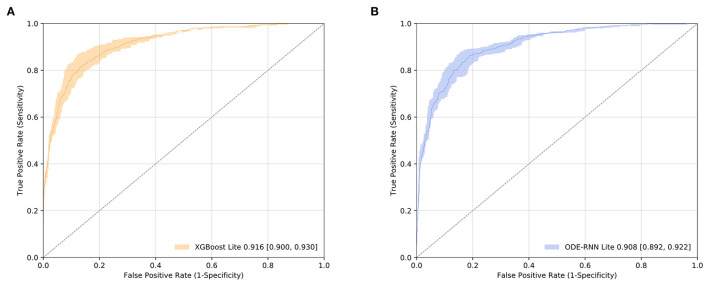
Lite model performance evaluation. **(A)** Receiver operating characteristic curves of XGBoost for DIC. **(B)** Receiver operating characteristic curves of ODE-RNN for DIC.

To enhance the transferability and reduce the data requirements of the model, we selected the ten most influential features based on SHAP values, Occlusion analysis, and clinical practicability (The Lite model feature details were shown in Supplementary File 2). Based on the selected features, we constructed the Lite model. Figure 8 indicated that the XGBoost-based Lite model achieves the best performance of predicting DIC 8 h in advance, but the model performance is slightly lower than that of the Full model (AUROC: 0.916 vs. 0.955).

## Discussion

In this study, we developed two models enabling real-time sequential monitoring of SIC and DIC disease progression in sepsis. The model could identify high-risk patients 48 h before the clinical diagnosis of SIC and sepsis-associated DIC, achieving the state-of-the-art retrospective performance. The full XGBoost model currently achieved the highest prediction performance (SIC: 0.973; DIC: 0.955). The Lite XGBoost model also achieved pleasing prediction performance (DIC: 0.916). On the BIDMC test set, the model performance decreased slightly (DIC: 0.865). In the 8 h prediction time-window with a threshold of 0.7, the XGBoost model was able to predict 89.5% of SIC and 83.1% of sepsis-associated DIC events correctly. Meanwhile, our study has introduced a methodological improvement. Specifically, our study provided the following contributions: (1) We developed and validated the first model for earlier sequential dynamic monitoring of sepsis-induced coagulation disease progression; (2) We processed irregular time-series data for the first time in dynamic prediction research using ODE method, achieving predictions at arbitrary time points; (3) We provided the visual interpretation for deep learning models and machine learning models, respectively, which improved the recognition of physicians toward complex models.

Sepsis-induced SIC, particularly sepsis-associated DIC, is a major cause of increased mortality in sepsis. However, clinical DIC diagnosis relies on FIB and D-Dimer whose laboratory testing frequency is <10% (31), resulting in a lag in DIC diagnosis. Therefore, it is meaningful to use a full spectrum of laboratory tests for the early detection of coagulation disorders. To our knowledge, there is only one study using machine learning to predict the progression of sepsis-induced DIC that was published in 2020 (32). Hasegawa et al. performed three classical machine learning methods to predict the progression of sepsis-induced coagulation disorders. In that study, sepsis was defined based on Systemic Inflammatory Response Syndrome (SIRS) criteria rather than sepsis 3.0 criteria. In addition, the study used the static data and the accuracy of the model to predict the progression of coagulopathy was only 59.8–67.0% (32). This is far poorer than our model prediction performance, which suggests that dynamic data monitoring is more consistent with clinical application than static models. In our study, high-risk patients were identified up to 48 h earlier, which suggests that comprehensive use of laboratory tests could detect coagulation disorders earlier. The Lite model also achieves satisfactory predictive performance.

The irregularity of clinical time series data was reflected in the tables as a large number of missing. Previous studies deal with large amounts of missing data by removing missing variables or using statistical interpolation methods, but such methods are not applicable to time series data (33). Neural ODE is a continuous dynamics theory that can explore the dynamic interactions between key features in the timeline of event onset and development (34). Our results demonstrate the superiority of ODE in the dynamics of disease. In a recent review, Alber et al. showed that it remains a challenge to apply ODE in medical continuous monitoring studies with incomplete baseline data and low-sampling data (35). Our study introduced ODE into RNNs and achieved better performance than RNN and LSTM, showing that ODE-RNNs are more appropriate for sparse irregular data than standard deep learning models. However, our results showed that the performance of the ODE-RNN was lower than that of the gradient boosted tree model (XGBoost and LightGBM). We consider that clinical laboratory diagnoses usually use hierarchical stratification for diagnosis, which fits better with the splitting structure of the tree model and gives the tree model a natural advantage. In addition, Qin et al. showed that neural models are not good at efficient feature transformation and scaling, while the tree-based model has an advantage in this respect (36). However, the flexibility and variety of tasks that can be achieved with deep learning are not available with traditional methods when dealing with complex tabular problems. Furthermore, occlusion analysis showed that a single feature masking does not affect the model significantly, indicating that ODE-RNN has better perturbation resistance. That is, the overall absence of a particular examination does not have a large impact on the ODE-RNN model, suggesting that the ODE-RNN model may have better robustness. Finally, the arbitrary time point continuous prediction achieved by ODE-RNN is also not possible with the gradient boosted tree model, where this arbitrary time point continuity is significant for clinical applications.

Our study offers the following potential benefits: Firstly, it is essential for ICU clinicians and nurses to identify patients who truly need intensive attention and personalized preventive medication. Our predictor can reduce the alarm frequency in patients without a high risk of sepsis-associated DIC occurrence after SIC. Secondly, our ODE-RNN model provides a reference for model selection of real-world irregular time series processing. This will facilitate subsequent studies to build robust models that better match the characteristics of clinical data. Thirdly, our model can be used to identify sepsis patients in different states of coagulation impairment, which could be useful for future randomized controlled clinical studies and further assist physicians to evaluate the time window of anticoagulation therapy appropriately. Finally, our interpretable model provides a visual interactive operating system prototype for early warning systems in ICU and will facilitate clinical deployment of predictive models.

However, some limitations also exist in our study. First of all, our model was developed in a single center, which reduces the effectiveness and may require retraining when the model is migrated to other centers. Furthermore, because we failed to obtain bedside real-time vital sign monitoring data, our model did not incorporate these parameters which may diminish the efficacy of the model. Finally, our study is retrospective and further prospective clinical studies need to be validated.

## Conclusion

Our early dynamic sequential predictor enables identification of sepsis patients at high risk of SIC and DIC up to 48 h earlier, achieving the highest performance to date. Our study showed that the ODE-RNN model achieves better performance than the standard RNN model. Our study contributes to early personalized management, and also improves the currently available algorithms.

## Data Availability Statement

The raw data supporting the conclusions of this article will be made available by the authors, without undue reservation.

## Ethics Statement

The studies involving human participants were reviewed and approved by XJTU1AF2020LSL-003. Written informed consent for participation was not required for this study in accordance with the national legislation and the institutional requirements.

## Author Contributions

RC designed the experiments, provided and preprocessed the clinical data, developed the supervised learning pipelines, and drafted the manuscript. WH designed the experiments, developed the machine learning pipelines, constructed the deep learning pipelines, and drafted the manuscript. KQ preprocessed the data and carried out the multivariate regression analysis. HY annotated the disease status labels and developed the deep learning pipelines. YT, QL, and HW carried out the data filtering and univariate analysis. YM, SL, and TL defined the clinically reportable range and performed multivariate regression analysis. JZ, JS, and CL revised the manuscript, conceived, and directed the project. All authors read and approved the final manuscript.

## Funding

This study was supported by the Critical Clinical Research Project of the First Affiliated Hospital of Xi'an Jiaotong University (No. XJTU1AF-CRF-2020-003) and the Joint Project of Universities in Shaanxi Province - Key Project (No. 2021GXLH-Z-099).

## Conflict of Interest

The authors declare that the research was conducted in the absence of any commercial or financial relationships that could be construed as a potential conflict of interest.

## Publisher's Note

All claims expressed in this article are solely those of the authors and do not necessarily represent those of their affiliated organizations, or those of the publisher, the editors and the reviewers. Any product that may be evaluated in this article, or claim that may be made by its manufacturer, is not guaranteed or endorsed by the publisher.
