# Supplementary material for: An Interpretable Early Dynamic Sequential Predictor for Sepsis-Induced Coagulopathy Progression in the Real-World Using Machine Learning
Source: Front Med (Lausanne). 2021 Dec 3;8:775047. doi: 10.3389/fmed.2021.775047 (PMC8678506; doi:10.3389/fmed.2021.775047)
Supplement: Supplementary file 1 [file Data_Sheet_1.docx]

**An interpretable early dynamic sequential predictor for sepsis-induced coagulopathy progression in the real-world using machine learning**

Electronic supplementary material

**Authors:**

Ruixia Cui^1,2^**^†^**, Wenbo Hua^3^**^†^**, Kai Qu^1^, Heran Yang^3^, Yingmu Tong^1,2^, Qinglin Li^1,2^, Hai Wang^1,2^, Yanfen Ma^4^, Sinan Liu^1,2^, Ting Lin^1,2^, Jingyao Zhang^1,2,5*^, Jian Sun^3*^, Chang Liu^1,3,5*^

^1^ Department of Hepatobiliary Surgery, The First Affiliated Hospital of Xi’an Jiaotong University, Xi’an, China.

^2^ Department of SICU, The First Affiliated Hospital of Xi’an Jiaotong University, Xi’an, China.

^3^ School of Mathematics and Statistics, Xi’an Jiaotong University, Xi’an, China.

^4^ Department of Clinical Laboratory, The First Affiliated Hospital of Xi’an Jiaotong University, Xi’an, China.

^5^ Biobank, The First Affiliated Hospital of Xi’an Jiaotong University, Xi’an, China.

**Corresponding author**: Chang Liu, Jian Sun and Jingyao Zhang

Correspondence to Jingyao Zhang, E-mail: Jingyaozhang@xjtu.edu.cn

Address: Department of SICU, The First Affiliated Hospital of Xi'an Jiaotong University, Xi'an, 710061, Shaanxi Province, China.

**Supplementary Method**

**Model construction Method**

We developed eight machine learning models for continuously predicting disease progression as main text. This study serves to predict SIC and sepsis-associated DIC from real-world time series data. The time-series data consists of two dimensions, a feature-dimension and a time-dimension. The machine learning methods used in this paper, such as LR, SVM, XGBoost, and LightGBM models require a one-dimensional vector as input, and therefore time-series data cannot be fed directly. Therefore, we compress the time-dimension into the feature-dimension to form a one-dimensional vector for training. In contrast, the deep learning methods such as RNN, LSTM, RNN-Decay, and ODE-RNN models, can directly process multivariate time-series without dimensional compression. The construction of machine learning models and standard RNN and LSTM models is well documented and will not be repeated in this study. The platforms used for their construction are described above. We will focus on the construction of RNN-Decay and ODE-RNN. The improved model architecture diagrams were shown in **Figure 3**

For the RNN-Decay model, we introduced a time decay mechanism based on the standard RNN to process irregular time series. The model incorporates a negative exponentially diminishing function in updating the hidden layer information $h_{n}$, denoted as equation (3). The information conveyed by the model gradually decays down as the time interval increases. We also added regressors for reconstructing the original data, and using the loss of mean square error at observable points to constrain the complexity of the model.

$h_{i}=RNNCell(h_{i-1}\cdot exp\{-\tau\Delta t\}, x_{i})$ (3)

For the ODE-RNN model, we introduced neural ODE into the standard RNN. The ODE is a series of continuous time models that define the hidden state $h_{(t)}$as the solution to the ODE initial value problem, as equation (4).

$\frac{dh(t)}{dt}=f(h(t), t,\theta) where h(t_{0})=h_{0}$ (4)

Where$f$is a dynamic representation of the hidden state with an initial value of$h_{0}$, a neural network with parameter 𝜃 is used to simulate$f$. The solution of the ODE allows to process the data at arbitrary time points and extract information. This allows us to continuity modelling the ODE solver (5) to extract the implicit information$h_{n}$in the irregular series and reduce the effect of data irregularity on the model output.

$h_{0}, . . . , h_{N}=ODESolve(f, h_{0},\left( t_{0}, . . . , t_{N} \right),\theta)$ (5)

$h_{i}^{'}=ODESolve(f, h_{i-1}, \left( t_{i-1},t_{i} \right))$ (6)

$h_{i}=RNNCell\left( h_{i}^{'},x_{i} \right)$ (7)

Unlike RNN-Decay, we feed the$h_{i}^{'}$calculated by the ODE solver into the RNN network to update the hidden layer states, which in turn use the$h_{n}$to predict the model output (equation (6)). Similarly, a regressor was introduced to extract the sequence output of the RNN to reconstruct original data (equation (7)).

**Supplementary Figures and Tables**

# Supplementary Figure 1. Demonstrations of temporal variable data missingness in XJTUMC and BIDMC

**

**

Each row represents a laboratory feature. x-axis, missing rate; y-axis, input feature.

In XJTUMC, the minimum rate of missing data is 62% and the maximum is 100%; In BIDMC, the minimum rate of missing data is 55% and the maximum is 100%.

# Supplementary Figure 2. Coagulation status annotation workflow


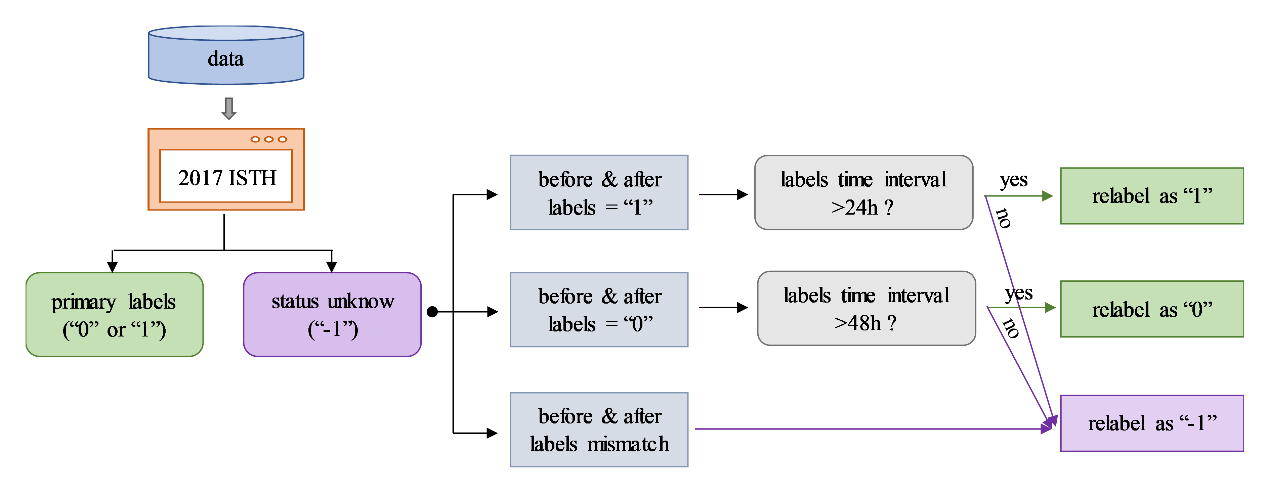


Three disease states are defined: "0" denotes a normal state, where the patient has not been diagnosed as SIC or overt-DIC; "1" denotes a diseased state, where the patient has been diagnosed as SIC or overt-DIC; "-1 " indicates an unknown status, where the status of the patient cannot be determined due to the absence of important features.

# Supplementary Table 1. SIC and ISTH overt-DIC criteria for coagulation status annotation

| **Step1: SIC onset lable (Score >= 4)** | |
| --- | --- |
| Platelet Count (K/u): | |
| <100 | point = 2 |
| >=100 and<150 | point = 1 |
| INR: | |
| >1.4 | point = 2 |
| >1.2 and <=1.4 | point = 1 |
| Adjust_SOFA: | |
| >=2 | point = 2 |
| 1 | point = 1 |
| **Step2: DIC onset lable (Score >= 4)** | |
| Platelet Count (K/ul): | |
| <50 | point = 2 |
| >=50 and <100 | point = 1 |
| D-Dimer (ng/mL): | |
| >=3000 and <7000 | point = 2 |
| >=7000 | point = 3 |
| Fibrinogen (mg/dL): | |
| <1000 | point = 1 |
| PT (s): | |
| >=19 | point = 2 |
| >=16 and <19 | point = 1 |

Note: Adjust_SOFA score is the sum of four items (respiratory SOFA, cardiovascular SOFA, hepatic SOFA, renal SOFA). SIC, sepsis-induced coagulopathy; DIC, disseminated intravascular coagulation; ISTH, International Society on Thrombosis and Hemostasis; INR, international normalization ratio; SOFA, sequential organ failure assessment; PT, prothrombin time.

# Supplementary Table 2. Pre-experiments for sampling time-window selection

| Model | SIC | | | DIC | | |
| --- | --- | --- | --- | --- | --- | --- |
| Sampling time-window | 8h | 24h | 48h | 8h | 24h | 48h |
| LR | 0.864 | 0.905 | 0.923 | 0.835 | 0.883 | 0.893 |
| SVM | 0.804 | 0.858 | 0.872 | 0.780 | 0.843 | 0.857 |
| XGBoost | 0.937 | 0.973 | 0.977 | 0.929 | 0.955 | 0.959 |
| LightGBM | 0.916 | 0.970 | 0.979 | 0.880 | 0.953 | 0.964 |
| RNN | 0.914 | 0.957 | 0.967 | 0.868 | 0.924 | 0.930 |
| LSTM | 0.916 | 0.961 | 0.971 | 0.862 | 0.928 | 0.941 |
| RNN-Decay |  | 0.950 | 0.962 |  | 0.927 | 0.943 |
| ODE-RNN |  | 0.962 | 0.969 |  | 0.936 | 0.957 |

Note: SIC, sepsis-induced coagulopathy; DIC, disseminated intravascular coagulation; LR, Logistic Regression; SVM, Support Vector Machines; LightGBM, Light Gradient Boosting Machine; XGBoost, eXtreme Gradient Boosting; RNN, Recurrent Neural Network; LSTM, Long Short-Term Memory network; RNN-Decay, Recurrent Neural Networks-Decay; ODE-RNN, Ordinary Differential Equations-Recurrent Neural Networks.

# Supplementary Table 3. Model performance for different warning thresholds 8h in advance for SIC prediction

| Models | Thresholds | Sensitivity | Specificity | PPV | NPV | F1 score |
| --- | --- | --- | --- | --- | --- | --- |
| LR | 0.5 | 0.934 | 0.647 | 0.879 | 0.782 | 0.906 |
|  | 0.7 | 0.834 | 0.827 | 0.930 | 0.645 | 0.880 |
|  | 0.9 | 0.575 | 0.960 | 0.975 | 0.451 | 0.724 |
| SVM | 0.5 | 0.921 | 0.582 | 0.858 | 0.730 | 0.889 |
|  | 0.7 | 0.849 | 0.743 | 0.901 | 0.642 | 0.874 |
|  | 0.9 | 0.385 | 0.941 | 0.948 | 0.358 | 0.547 |
| XGBoost | 0.5 | 0.965 | 0.809 | 0.933 | 0.894 | 0.949 |
|  | 0.7 | 0.905 | 0.937 | 0.975 | 0.781 | 0.939 |
|  | 0.9 | 0.659 | 0.988 | 0.993 | 0.513 | 0.792 |
| LightGBM | 0.5 | 0.988 | 0.602 | 0.872 | 0.947 | 0.926 |
|  | 0.7 | 0.984 | 0.661 | 0.888 | 0.937 | 0.934 |
|  | 0.9 | 0.970 | 0.770 | 0.921 | 0.904 | 0.945 |
| RNN | 0.5 | 0.948 | 0.763 | 0.916 | 0.843 | 0.932 |
|  | 0.7 | 0.911 | 0.867 | 0.950 | 0.781 | 0.930 |
|  | 0.9 | 0.851 | 0.938 | 0.974 | 0.697 | 0.909 |
| LSTM | 0.5 | 0.917 | 0.864 | 0.949 | 0.791 | 0.933 |
|  | 0.7 | 0.868 | 0.934 | 0.973 | 0.721 | 0.918 |
|  | 0.9 | 0.802 | 0.966 | 0.985 | 0.640 | 0.884 |
| RNN-Decay | 0.5 | 0.918 | 0.820 | 0.933 | 0.784 | 0.925 |
|  | 0.7 | 0.864 | 0.901 | 0.960 | 0.707 | 0.910 |
|  | 0.9 | 0.765 | 0.969 | 0.986 | 0.601 | 0.862 |
| ODE-RNN | 0.5 | 0.923 | 0.841 | 0.941 | 0.798 | 0.932 |
|  | 0.7 | 0.886 | 0.920 | 0.968 | 0.745 | 0.925 |
|  | 0.9 | 0.815 | 0.966 | 0.985 | 0.655 | 0.892 |

Note: PPV, Positive predictive value; NPV, Negative predictive value. LR, Logistic Regression; SVM, Support Vector Machines; LightGBM, Light Gradient Boosting Machine; XGBoost, eXtreme Gradient Boosting; RNN, Recurrent Neural Network; LSTM, Long Short-Term Memory network; RNN-Decay, Recurrent Neural Networks-Decay; ODE-RNN, Ordinary Differential Equations-Recurrent Neural Networks.

# Supplementary Table 4. Model performance for different warning thresholds 8h in advance for DIC prediction

| Models | Thresholds | Sensitivity | Specificity | PPV | NPV | F1 score |
| --- | --- | --- | --- | --- | --- | --- |
| LR | 0.5 | 0.704 | 0.892 | 0.802 | 0.829 | 0.750 |
|  | 0.7 | 0.517 | 0.969 | 0.912 | 0.763 | 0.660 |
|  | 0.9 | 0.311 | 0.992 | 0.959 | 0.699 | 0.470 |
| SVM | 0.5 | 0.548 | 0.915 | 0.800 | 0.765 | 0.650 |
|  | 0.7 | 0.442 | 0.950 | 0.845 | 0.733 | 0.580 |
|  | 0.9 | 0.071 | 0.999 | 0.977 | 0.634 | 0.133 |
| XGBoost | 0.5 | 0.902 | 0.870 | 0.812 | 0.935 | 0.855 |
|  | 0.7 | 0.760 | 0.968 | 0.937 | 0.866 | 0.839 |
|  | 0.9 | 0.258 | 0.999 | 0.994 | 0.684 | 0.410 |
| LightGBM | 0.5 | 0.967 | 0.641 | 0.626 | 0.969 | 0.760 |
|  | 0.7 | 0.949 | 0.742 | 0.695 | 0.959 | 0.803 |
|  | 0.9 | 0.892 | 0.880 | 0.822 | 0.929 | 0.856 |
| RNN | 0.5 | 0.699 | 0.935 | 0.870 | 0.833 | 0.775 |
|  | 0.7 | 0.596 | 0.962 | 0.907 | 0.793 | 0.719 |
|  | 0.9 | 0.363 | 0.988 | 0.948 | 0.714 | 0.525 |
| LSTM | 0.5 | 0.700 | 0.948 | 0.892 | 0.836 | 0.785 |
|  | 0.7 | 0.568 | 0.972 | 0.927 | 0.784 | 0.704 |
|  | 0.9 | 0.356 | 0.990 | 0.956 | 0.712 | 0.519 |
| RNN-Decay | 0.5 | 0.752 | 0.918 | 0.850 | 0.856 | 0.798 |
|  | 0.7 | 0.629 | 0.952 | 0.890 | 0.805 | 0.737 |
|  | 0.9 | 0.437 | 0.984 | 0.943 | 0.738 | 0.597 |
| ODE-RNN | 0.5 | 0.801 | 0.910 | 0.848 | 0.881 | 0.824 |
|  | 0.7 | 0.700 | 0.944 | 0.887 | 0.835 | 0.783 |
|  | 0.9 | 0.512 | 0.972 | 0.920 | 0.762 | 0.657 |

Note: PPV, Positive predictive value; NPV, Negative predictive value. LR, Logistic Regression; SVM, Support Vector Machines; LightGBM, Light Gradient Boosting Machine; XGBoost, eXtreme Gradient Boosting; RNN, Recurrent Neural Network; LSTM, Long Short-Term Memory network; RNN-Decay, Recurrent Neural Networks-Decay; ODE-RNN, Ordinary Differential Equations-Recurrent Neural Networks.

# Supplementary Table 5. Logistic univariate and multivariate regression analysis of SIC onset

|  | Univariate analysis  OR (95%CI) | p-value | Multivariate analysis  OR (95%CI) | p-value |
| --- | --- | --- | --- | --- |
| Baseline characteristics | | | | |
| Male | 0.926 (0.808, 1.062) | 0.271 |  |  |
| Age (year) | 1.021 (1.016, 1.025) | < 0.001 | 1.006 (0.998, 1.013) | 0.167 |
| SOFA | 1.730 (1.636, 1.830) | < 0.001 | 1.223 (1.123, 1.332) | **< 0.001** |
| Infection sources | 1.009 (0.964, 1.055) | 0.700 |  |  |
| Laboratory Test | | | | |
| RBC (10^12^/L) | 0.464 (0.420, 0.513) | < 0.001 | 0.094 (0.023, 0.393) | **0.001** |
| WBC (10^9^/L) | 1.046 (1.034, 1.058) | < 0.001 | 1.011 (0.987, 1.035) | 0.367 |
| NEUT (%) | 1.047 (1.041, 1.054) | < 0.001 | 1.028 (1.003, 1.054) | **0.028** |
| LYMPH (%) | 0.957 (0.950, 0.964) | < 0.001 | 1.032 (1.002, 1.062) | **0.034** |
| EO (%) | 0.983 (0.856, 1.128) | 0.806 | 0.02 [0.00, 0.06] |  |
| HGB (g/dL) | 0.810 (0.785, 0.836) | < 0.001 | 2.124 (1.335, 3.381) | **0.001** |
| HCT (%) | 0.928 (0.920, 0.937) | < 0.001 | 0.982 (0.953, 1.012) | 0.23 |
| MCV (fL) | 1.038 (1.028, 1.049) | < 0.001 | 1.034 (0.982, 1.089) | 0.206 |
| MCH (pg) | 1.086 (1.058, 1.116) | < 0.001 | 0.715 (0.594, 0.861) | **< 0.001** |
| RDW-SD (fL) | 1.080 (1.068, 1.093) | < 0.001 | 0.996 (0.932, 1.064) | 0.901 |
| RDW-CV (%) | 1.281 (1.230, 1.334) | < 0.001 | 1.233 (1.003, 1.515) | **0.046** |
| PCT (‰) | 0.138 (0.120, 0.160) | < 0.001 | 0.239 (0.200, 0.285) | **< 0.001** |
| P-LCR (%) | 1.060 (1.053, 1.068) | < 0.001 | 1.035 (1.023, 1.046) | **< 0.001** |
| ALB (g/L) | 0.882 (0.871, 0.893) | < 0.001 | 0.960 (0.936, 0.985) | **0.002** |
| CHOI (mg/dL) | 0.983 (0.981, 0.984) | < 0.001 | 0.993 (0.991, 0.996) | **< 0.001** |
| BUN (mg/dL) | 1.058 (1.051, 1.065) | < 0.001 | 1.018 (1.009, 1.028) | **< 0.001** |
| Glu (mg/dL) | 1.004 (1.003, 1.005) | < 0.001 | 1.001 (0.999, 1.003) | 0.408 |
| Na (mmol/L) | 0.936 (0.823, 0.948) | < 0.001 | 0.983 (0.959, 1.007) | 0.168 |
| Ca (mg/dL) | 0.380 (0.342, 0.423) | < 0.001 | 0.829 (0.683, 1.007) | 0.058 |

Note: In the univariate and multifactorial analysis, we excluded coagulation-related indicators and SOFA score-related indicators, as these have been used to diagnose SIC and DIC.

# Supplementary Table 6. Logistic univariate and multivariate regression analysis of DIC onset

|  | Univariate analysis  OR (95%CI) | p-value | Multivariate analysis  OR (95%CI) | p-value |
| --- | --- | --- | --- | --- |
| Baseline characteristics | | | | |
| Male | 0.909 (0.765, 1.081) | 0.281 |  |  |
| Age (year) | 1.018 (1.012, 1.023) | < 0.001 | 1.002 (0.993, 1.010) | 0.702 |
| SOFA | 1.838 (1.737, 1.946) | < 0.001 | 1.367 (1.266, 1.477) | **< 0.001** |
| Infection sources | 1.087 (1.030, 1.148) | 0.002 |  |  |
| Laboratory Test | | | | |
| RBC (10^12^/L) | 0.431 (0.383, 0.486) | < 0.001 | 0.087 (0.019, 0.407) | **0.002** |
| WBC (10^9^/L) | 1.078 (1.065, 1.092) | < 0.001 | 1.027 (1.005, 1.050) | **0.017** |
| NEUT (%) | 1.064 (1.055, 1.074) | < 0.001 | 1.000 (0.974, 1.027) | 0.995 |
| LYMPH (%) | 0.939 (0.929, 0.950) | < 0.001 | 1.016 (0.985, 1.047) | 0.321 |
| EO (%) | 0.968 (0.795, 1.180) | 0.750 |  |  |
| HGB (g/dL) | 0.776 (0.746, 0.806) | < 0.001 | 2.512 (1.515, 4.163) | **0.001** |
| HCT (%) | 0.916 (0.905, 0.927) | < 0.001 | 0.964 (0.927, 1.001) | 0.057 |
| MCV (fL) | 1.023 (1.010, 1.036) | < 0.001 | 1.074 (1.019, 1.133) | **0.008** |
| MCH (pg) | 1.048 (1.015, 1.081) | 0.004 | 0.665 (0.546, 0.810) | **< 0.001** |
| RDW-SD (fL) | 1.064 (1.051, 1.077) | < 0.001 | 0.950 (0.893, 1.011) | 0.106 |
| RDW-CV (%) | 1.265 (1.214, 1.318) | < 0.001 | 1.429 (1.178, 1.734) | **< 0.001** |
| PCT (‰) | 0.335 (0.295, 0.380) | < 0.001 | 0.663 (0.566, 0.776) | **< 0.001** |
| P-LCR (%) | 1.033 (1.025, 1.041) | < 0.001 | 1.004 (0.992, 1.017) | 0.510 |
| ALB (g/L) | 0.854 (0.841, 0.868) | < 0.001 | 0.949 (0.924, 0.976) | **< 0.001** |
| CHOI (mg/dL) | 0.977 (0.974, 0.979) | < 0.001 | 0.991 (0.988, 0.994) | **< 0.001** |
| BUN (mg/dL) | 1.053 (1.047, 1.059) | < 0.001 | 1.016 (1.008, 1.024) | **< 0.001** |
| Glu (mg/dL) | 1.004 (1.003, 1.005) | < 0.001 | 1.000 (0.998, 1.002) | 0.776 |
| Na (mmol/L) | 0.937 (0.922, 0.951) | < 0.001 | 0.989 (0.964, 1.014) | 0.381 |
| Ca (mg/dL) | 0.352 (0.310, 0.399) | < 0.001 | 0.659 (0.537, 0.810) | **< 0.001** |

Note: In the univariate and multifactorial analysis, we excluded coagulation-related indicators and SOFA score-related indicators, as these have been used to diagnose SIC and DIC.
