# Supplementary material for: An Interpretable Early Dynamic Sequential Predictor for Sepsis-Induced Coagulopathy Progression in the Real-World Using Machine Learning
Source: Front Med (Lausanne). 2021 Dec 3;8:775047. doi: 10.3389/fmed.2021.775047 (PMC8678506; doi:10.3389/fmed.2021.775047)
Supplement: Supplementary file 2 [file Data_Sheet_2.DOCX]

**Supplemental File 2**

Available laboratory variables and Clinical reportable ranges at XJTUMC and BIDMC

| Test _category | MIMIC_itemid | Variable_name | XJTU_defabb | Permitted range | Unit | Lite |
| --- | --- | --- | --- | --- | --- | --- |
| Blood Gas | 50801 | Alveolar-arterial Gradient | PA-aDO2 | [10,50] | mmHg |  |
| Blood Gas | 50802 | Base Excess | ABE | [-30,30] | mmol/L |  |
| Blood Gas | 50804 | Calculated Total CO2 | TCO2 | [10,50] | mmol/L |  |
| Blood Gas | 50805 | Carboxyhemoglobin | FCOHb | [0,27] | g/dL |  |
| Blood Gas | 50806 | Chloride, Whole Blood | Cl- | [60,150] | mmol/L |  |
| Blood Gas | 50808 | Free Calcium | Ca2+ | [0.2,5] | mmol/L |  |
| Blood Gas | 50809 | Glucose | gGlu | [20,625] | mg/dL |  |
| Blood Gas | 50810 | Hematocrit, Calculated | HCT | [0,75] | % | **√** |
| Blood Gas | 50811 | Hemoglobin | gHGB | [2,27] | g/dL |  |
| Blood Gas | 50813 | Lactate | Lac | [0,15] | mmol/L |  |
| Blood Gas | 50814 | Methemoglobin | MetHb | [0,27] | g/dL |  |
| Blood Gas | 50817 | Oxygen Saturation | SpO2 | [10,100] | % |  |
| Blood Gas | 50818 | pCO2 | pCO2 | [5,250] | mmHg |  |
| Blood Gas | 50820 | pH | pH | [6.0,8.0] |  |  |
| Blood Gas | 50821 | pO2 | pO2 | [20,500] | mmHg | **√** |
| Blood Gas | 50822 | Potassium, Whole Blood | K+ | [2,8] | mmol/L |  |
| Blood Gas | 50824 | Sodium, Whole Blood | Na+ | [100,170] | mmol/L |  |
| Blood Gas | 50825 | Temperature | T | [32,45] | °C |  |
| Chemistry | 50852 | % Hemoglobin A1c | HBA1c | [0.36,12.3] | % |  |
| Chemistry | 50861 | Alanine Aminotransferase | ALT | [3,15680] | U/L |  |
| Chemistry | 50862 | Albumin | ALB | [10,70] | g/L |  |
| Chemistry | 50863 | Alkaline Phosphatase | ALP | [2,12000] | U/L |  |
| Chemistry | 50867 | Amylase | AMY | [4,1200] | U/L |  |
| Chemistry | 50868 | Anion Gap | AG | [0,30] | mmol/L |  |
| Chemistry | 50878 | Asparate Aminotransferase | AST | [2,11550] | U/L |  |
| Blood Gas | 50882 | Bicarbonate | AB | [0,50] | mmol/L |  |
| Chemistry | 50883 | Bilirubin, Direct | DBIL | [0,28] | mg/dL |  |
| Chemistry | 50884 | Bilirubin, Indirect | IDBIL | [0,28] | mg/dL |  |
| Chemistry | 50885 | Bilirubin, Total | TBIL | [0.1,28] | mg/dL |  |
| Chemistry | 50893 | Calcium, Total | Ca | [1,15] | mg/dL |  |
| Chemistry | 50902 | Chloride | Cl | [50,175] | mmol/L |  |
| Chemistry | 50904 | Cholesterol, HDL | HDL | [1,500] | mg/dL |  |
| Chemistry | 50906 | Cholesterol, LDL, Measured | LDL | [4,1200] | mg/dL |  |
| Chemistry | 50907 | Cholesterol, Total | CHOI | [10,2000] | mg/dL | **√** |
| Chemistry | 50910 | Creatine Kinase | CK | [2,16000] | U/L |  |
| Chemistry | 50911 | Creatine Kinase, MB Isoenzyme | CK-MB | [0,2000] | U/L |  |
| Chemistry | 50912 | Creatinine | CRE | [0,500] | mg/dL |  |
| Chemistry | 50927 | Gamma Glutamyltransferase | GGT | [0,2500] | U/L |  |
| Chemistry | 50930 | Globulin | GLB | [0,11] | g/dL |  |
| Chemistry | 50931 | Glucose | Glu | [20,625] | mg/dL |  |
| Chemistry | 50954 | Lactate Dehydrogenase | LDH | [4,5000] | U/L |  |
| Chemistry | 50960 | Magnesium | Mg | [0.5,5] | mg/dL |  |
| Chemistry | 50963 | NTproBNP | proBNP | [0,5000] | pg/mL |  |
| Chemistry | 50970 | Phosphate | P | [0.5,13] | mg/dL |  |
| Chemistry | 50971 | Potassium | K | [1,14] | mmol/L |  |
| Chemistry | 50976 | Protein, Total | TP | [2,11] | g/dL |  |
| Chemistry | 50983 | Sodium | Na | [75,250] | mmol/L |  |
| Chemistry | 51000 | Triglycerides | TG | [10,6000] | mg/dL |  |
| Chemistry | 51003 | Troponin T | TnT | [0,1.5] | ng/mL |  |
| Chemistry | 51006 | Urea Nitrogen | BUN | [2,126] | mg/dL | **√** |
| Chemistry | 51007 | Uric Acid | UA | [0.5,50] | mg/dL |  |
| Hematology | 51146 | Basophils | BASO% | [0,10] | % |  |
| Hematology | 51196 | D-Dimer | D-Dimer | [0,10000] | ng/mL |  |
| Hematology | 51199 | Eosinophil | EO% | [0,10] | % |  |
| Hematology | 51213 | Fibrin Degradation Products | FDP | [0,50] | mg/L |  |
| Hematology | 51214 | Fibrinogen, Functional | FIB | [50,4500] | mg/dL | **√** |
| Hematology | 51222 | Hemoglobin | HGB | [2,25] | g/dL | **√** |
| Hematology | 51237 | INR | INR | [0,8] |  | **√** |
| Hematology | 51245 | Lymphocytes, Percent | LYMPH% | [0,100] | % |  |
| Hematology | 51248 | MCH | MCH | [10,80] | pg |  |
| Hematology | 51249 | MCHC | MCHC | [100,600] | g/L |  |
| Hematology | 51250 | MCV | MCV | [50,180] | fL |  |
| Hematology | 51254 | Monocytes | MONO% | [0,30] | % |  |
| Hematology | 51256 | Neutrophils | NEUT% | [0,100] | % |  |
| Blood Gas | 51257 | Nucleated Red Cells | NRBC% | [0,6] | % |  |
| Hematology | 51265 | Platelet Count | PLT | [0,1000] | 10^9/L | **√** |
| Hematology | 51274 | PT | PT | [0,100] | s | **√** |
| Hematology | 51275 | PTT | APTT | [0,300] | s |  |
| Hematology | 51277 | RDW-CV | RDW-CV | [0,30] | % |  |
| Hematology | 51279 | Red Blood Cells | RBC | [0,8.6] | 10^12/L |  |
| Hematology | 51280 | Reptilase Time | TT | [0,100] | s |  |
| Hematology | 51300 | WBC Count | WBC | [0,100] | 10^9/L | **√** |
| Hematology |  | RDW-SD | RDW-SD | [20,80] | fL |  |
| Hematology |  | Carbondioxide Combining Power | CO2CP | [0,50] | mmol/L |  |
| Hematology |  | Mean Platelet Volume | MPV | [3,20] | fL |  |
| Hematology |  | Plateletcrit | PCT | [0,5] | % |  |
| Hematology |  | Platelet Distribution Width | PDW | [5,25] | fL |  |
| Hematology |  | Platelet -Larger Cell Ratio | P-LCR | [0,80] | % |  |
| Chemistry |  | Albumin-Globulin Ratio | A/G | [1,3] |  |  |
| Chemistry |  | Cystatin C | Cys-c | [0,200] | mg/L |  |
| Hematology |  | Immature Granulocyte | IG% | [0,10] | % |  |
| Chemistry |  | Procalcitonin | procalcitonin | [0,1000] | ng/mL |  |
| Chemistry |  | Bile Acids, Total | TBA | [0,5000] | umol/L |  |
| Chemistry |  | Superoxide Dismutase | SOD | All | U/L |  |
| Hematology |  | prothrombin time activity | PTA | [60,180] | % |  |
| Chemistry |  | Prealbumin | PA | [0,2000] | mg/L |  |
| Chemistry |  | Cholinesterase | CHE | All | U/L |  |
| Chemistry |  | α-Hydroxybutyrate Dehydrogenase | α-HBDH | [20,2000] | U/L |  |
| Chemistry |  | Oxygen Half-saturation Pressure of Hemoglobin | p50 | [20,60] | mmHg |  |
| Chemistry |  | G-lipopolysaccharides | LPS | [0,100] | pg/mL |  |
| Chemistry |  | Glycated Albumin | GA% | [0,35] | % |  |
| Chemistry |  | eGFR | eGFR | [10,150] | mL/min/1.73m^2^ |  |
| Chemistry |  | Apolipoprotein A | APOA | [0.03,6.8] | g/L |  |
| Chemistry |  | Apolipoprotein B | APOB | [0.04,6.9] | g/L |  |
| Chemistry |  | Apolipoprotein E | APOE | [10,100] | mg/L |  |
| Chemistry |  | Lipoprotein(a) | Lp(a) | [0,1200] | mg/L |  |
| Blood Gas |  | Oxyhemoglobin | FO2Hb | [0,27] | g/dL |  |
| Blood Gas |  | Deoxygenated Hemoglobin | HHb | [0,27] | g/dL |  |
| Blood Gas |  | Arterial Oxygen Content | CaO2 | [10,100] | mmol/L |  |

Note: MIMIC_itemid means the BIDMC laboratory test identifier; Variable_name refers to the generic name of the test at both centres; XJTU_defabb means the laboratory test abbreviation provided by XJTUMC; Permitted range is the range of results that can be reported to the clinic for a given quantitative test; The units of test data from the two central laboratories have been converted and standardized.
