# Supplementary material for: An Interpretable Early Dynamic Sequential Predictor for Sepsis-Induced Coagulopathy Progression in the Real-World Using Machine Learning
Source: Front Med (Lausanne). 2021 Dec 3;8:775047. doi: 10.3389/fmed.2021.775047 (PMC8678506; doi:10.3389/fmed.2021.775047)
Supplement: Supplementary file 3 [file Data_Sheet_3.DOCX]

**Supplemental File 3**

Occlusion analysis examines the impact of features on deep learning model predictions

| Variable_name | XJTU_defabb | SIC_AUROC | DIC_AUROC |
| --- | --- | --- | --- |
| D-Dimer | D-Dimer | 0.962 | 0.917 |
| Plateletcrit | PCT | 0.908 | 0.926 |
| Oxygen Half-saturation Pressure of Hemoglobin | p50 | 0.962 | 0.928 |
| Bilirubin, Indirect | IDBIL | 0.962 | 0.928 |
| Fibrin Degradation Products | FDP | 0.961 | 0.928 |
| Chloride | Cl | 0.963 | 0.931 |
| Fibrinogen, Functional | FIB | 0.961 | 0.931 |
| Bilirubin, Direct | DBIL | 0.963 | 0.931 |
| Monocytes | MONO% | 0.963 | 0.931 |
| prothrombin time activity | PTA | 0.961 | 0.931 |
| Urea Nitrogen | BUN | 0.961 | 0.932 |
| Sodium | Na | 0.962 | 0.932 |
| Platelet Count | PLT | 0.942 | 0.932 |
| Calcium, Total | Ca | 0.959 | 0.933 |
| Hematocrit, Calculated | HCT | 0.963 | 0.933 |
| Neutrophils | NEUT% | 0.962 | 0.933 |
| Uric Acid | UA | 0.961 | 0.933 |
| Globulin | GLB | 0.961 | 0.933 |
| Bilirubin, Total | TBIL | 0.962 | 0.933 |
| Procalcitonin | procalcitonin | 0.961 | 0.933 |
| MCH | MCH | 0.962 | 0.933 |
| Superoxide Dismutase | SOD | 0.963 | 0.934 |
| Amylase | AMY | 0.962 | 0.934 |
| Immature Granulocyte | IG% | 0.962 | 0.934 |
| Cholesterol, Total | CHOI | 0.958 | 0.934 |
| Lactate | Lac | 0.961 | 0.934 |
| MCV | MCV | 0.961 | 0.934 |
| Anion Gap | AG | 0.963 | 0.934 |
| Glucose | gGlu | 0.963 | 0.934 |
| Platelet Distribution Width | PDW | 0.962 | 0.934 |
| Creatine Kinase, MB Isoenzyme | CK-MB | 0.962 | 0.934 |
| Potassium | K | 0.961 | 0.934 |
| Albumin-Globulin Ratio | A/G | 0.962 | 0.934 |
| pH | pH | 0.961 | 0.934 |
| Lymphocytes, Percent | LYMPH% | 0.961 | 0.934 |
| NTproBNP | proBNP | 0.962 | 0.934 |
| Cystatin C | Cys-c | 0.962 | 0.934 |
| PT | PT | 0.960 | 0.935 |
| Platelet -Larger Cell Ratio | P-LCR | 0.961 | 0.935 |
| α-Hydroxybutyrate Dehydrogenase | α-HBDH | 0.96 | 0.93 |
| Alveolar-arterial Gradient | PA-aDO2 | 0.962 | 0.935 |
| Glucose | Glu | 0.959 | 0.935 |
| INR | INR | 0.961 | 0.935 |
| Oxygen Saturation | SpO2 | 0.962 | 0.935 |
| Magnesium | Mg | 0.962 | 0.935 |
| Asparate Aminotransferase | AST | 0.961 | 0.935 |
| Creatinine | CRE | 0.961 | 0.935 |
| Base Excess | ABE | 0.963 | 0.935 |
| Calculated Total CO2 | TCO2 | 0.963 | 0.935 |
| Bile Acids,Total | TBA | 0.961 | 0.935 |
| PTT | APTT | 0.960 | 0.935 |
| Alkaline Phosphatase | ALP | 0.962 | 0.935 |
| Cholinesterase | CHE | 0.962 | 0.935 |
| MCHC | MCHC | 0.962 | 0.935 |
| Mean Platelet Volume | MPV | 0.957 | 0.935 |
| Eosinophil | EO% | 0.963 | 0.936 |
| G-lipopolysaccharides | LPS | 0.962 | 0.936 |
| Lactate Dehydrogenase | LDH | 0.962 | 0.936 |
| Protein, Total | TP | 0.962 | 0.936 |
| Reptilase Time | TT | 0.962 | 0.936 |
| Gamma Glutamyltransferase | GGT | 0.963 | 0.936 |
| Estimated Glomerular Filtration Rate | eGFR | 0.962 | 0.936 |
| Red Blood Cells | RBC | 0.963 | 0.936 |
| Phosphate | P | 0.963 | 0.936 |
| Carbondioxide Combining Power | CO2CP | 0.963 | 0.936 |
| RDW-SD | RDW-SD | 0.961 | 0.936 |
| Free Calcium | Ca2+ | 0.963 | 0.936 |
| WBC Count | WBC | 0.962 | 0.936 |
| RDW-CV | RDW-CV | 0.962 | 0.936 |
| Arterial Oxygen Content | CaO2 | 0.962 | 0.936 |
| pCO2 | pCO2 | 0.962 | 0.936 |
| Glycated Albumin | GA% | 0.962 | 0.936 |
| Methemoglobin | MetHb | 0.962 | 0.936 |
| Carboxyhemoglobin | FCOHb | 0.962 | 0.936 |
| Oxyhemoglobin | FO2Hb | 0.962 | 0.936 |
| Apolipoprotein A | APOA | 0.962 | 0.936 |
| Deoxygenated Hemoglobin | HHb | 0.962 | 0.936 |
| pO2 | pO2 | 0.963 | 0.936 |
| Lipoprotein(a) | Lp(a) | 0.962 | 0.936 |
| Nucleated Red Cells | NRBC% | 0.962 | 0.936 |
| Hemoglobin | gHGB | 0.964 | 0.936 |
| Sodium, Whole Blood | Na+ | 0.961 | 0.936 |
| % Hemoglobin A1c | HBA1c | 0.962 | 0.936 |
| Hemoglobin | HGB | 0.964 | 0.936 |
| Cholesterol, LDL, Measured | LDL | 0.962 | 0.936 |
| Creatine Kinase | CK | 0.962 | 0.936 |
| Albumin | ALB | 0.962 | 0.936 |
| Triglycerides | TG | 0.962 | 0.936 |
| Troponin T | TnT | 0.962 | 0.936 |
| Prealbumin | PA | 0.963 | 0.936 |
| Cholesterol, HDL | HDL | 0.963 | 0.936 |
| Apolipoprotein B | APOB | 0.962 | 0.936 |
| Chloride, Whole Blood | Cl- | 0.962 | 0.936 |
| Alanine Aminotransferase | ALT | 0.962 | 0.936 |
| Apolipoprotein E | APOE | 0.962 | 0.936 |
| Basophils | BASO% | 0.962 | 0.937 |
| Bicarbonate | AB | 0.956 | 0.937 |
| Temperature | T | 0.962 | 0.937 |
| Potassium, Whole Blood | K+ | 0.960 | 0.937 |

Note: Occlusion analysis masked one feature by one. The greater alteration in AUROC after masking indicates a significant effect of the feature on the model output. The decrease in AUROC indicates that the feature has a positive effect on the model output and increase in AUROC indicates a negative effect of the feature on the model output.
